# Patients’ Perspectives of Surgical Safety Before and After Their Elective Surgeries at King Abdulaziz University Hospital, Jeddah, Saudi Arabia

**DOI:** 10.7759/cureus.6171

**Published:** 2019-11-16

**Authors:** Hatim A Al-Abbadi, Hadeel A Basharaheel, Maram R Alharbi, Hanin A Alharbi, Dalia Sindi, Marwa Bamatraf

**Affiliations:** 1 Surgery, King Abdulaziz University Hospital, Jeddah, SAU; 2 General Surgery, King Abdulaziz University Hospital, Jeddah, SAU

**Keywords:** general surgery, patient safety, jeddah, saudi arabia, surgical patients

## Abstract

Background

Patients need to be educated and all possible treatment alternatives should be explored. Patients should be given options that they can choose from based on their demographic information, clinical information, and possible options for treating a given issue. This is especially true in elective surgery. The concept of safety plays a major part in every field, particularly in medicine. The patient’s safety is a key factor for a better experience and a better outcome.

Objective

This study aims to determine patient perceptions of surgical safety with an emphasis on surgical team interaction throughout the phases of care.

Methods

A descriptive cross-sectional prospective study was conducted at King Abdulaziz University Hospital, Jeddah, Saudi Arabia. Patients undergoing elective surgery and hospitalized for more than 24 hours were asked to give their opinions regarding interactions between them and the surgical teams, including the nurses, anesthesiologists, and surgeons who operated on them. Only patients aged 18 or above were included in the sample. The analysis was carried out using the IBM Statistical Package for Social Sciences (SPSS), version 25 (IBM SPSS Statistics, Armonk, NY).

Results

More than 70% of the study respondents had had more than one surgery. One hundred and ten of the study respondents said that the specific surgeons who attended to them encouraged them to ask questions. The majority of the respondents (76.7%) said that the surgical team gave them definite physical comfort, while the rest (23.3%) stated that they got somewhat less physical comfort from the surgical team. The average rating of the satisfaction pre-surgery was mean (M) = 8.51, standard deviation (SD) = 1.9, (95% confidence interval (CI): 8.19 - 8.83) while the average satisfaction rating for postoperative care was M = 9.05, SD = 1.35, (95% CI: 8.82 - 9.27).

Conclusions

Most patients valued surgeon-patient interaction as it was seen to reduce pre-surgery anxiety, helped in giving options, and improved the patient’s overall understanding of the surgical procedure. Surgical teams are generally highly rated in terms of overall service pre- and post-surgery.

## Introduction

Surgery is an integral part of healthcare throughout the world, with an estimated 234 million operations performed annually [1]. In 2008, the World Health Organization (WHO) published guidelines identifying multiple recommended practices to ensure the safety of surgical patients worldwide [2]. Although surgical care can prevent loss of life or limb, it is also associated with a considerable risk of complications and death [3]. Surgical safety initiatives, including time-out, surgical safety checklists (SSCs), and debriefings, have been shown to reduce errors and improve outcomes [4]. Many of these processes require repetitious questioning of patients through different phases of care and, if misinterpreted, may be seen as a failure of communication or as provider incompetence, leading to dissatisfaction with the surgical experience [5]. Many factors influence patient satisfaction, and perceptions of safety may have a prominent role [6]. To make a surgical procedure successful, preparations should cover the materials and logistical issues required to successfully perform the surgery, the psychological preparation of the patient (e.g., anxiety), and explore all options that suit a patient’s economic ability and medical history which are important [7]. 

Patient satisfaction has become the main point of focus for the provision of not only the surgery service but also as a method of quality of care and medical/surgical intervention. Patients should be educated with all possible alternatives explored and should be given options that they can choose from based on their demographic information, clinical information, and possible options for treating a given issue [8]. When such information is provided to the patients, non-surgical options may not be used [9]. To increase the satisfaction of patients, their safety perceptions of surgery need to be defined. This study aimed at determining patient perceptions of surgical safety with an emphasis on surgical team interaction throughout the phases of care.

## Materials and methods

This study was approved by the Institutional Review Board of King Abdulaziz University (KAU), Jeddah, Saudi Arabia. A descriptive cross-sectional prospective study was conducted in the present case, where patients hospitalized for more than 24 hours and after an elective surgery were asked to give their opinions regarding interactions between them and the surgical teams, including nurses, anesthesiologists, and the surgeons who operated on them. Questionnaires were used as the main data collection tool where questions were read to the patients and the responses given to the researcher. The study was conducted at King Abdulaziz University Hospital, Jeddah, Saudi Arabia from June 2018 to December 2018. The target sample size was 100 patients, but 150 patients completed the survey questionnaire. Only patients aged 18 or above were included in the sample. The analysis was carried out using IBM Statistical Package for Social Sciences (SPSS), version 25 (IBM SPSS Statistics, Armonk, NY).

Consent and ethical issues

Patients were informed of the importance of the information given to them and only participated in the study willingly. Confidentiality was maintained by not allowing third-party access to information gathered and only using information gathered for research purposes strictly.

## Results

One hundred and fifty respondents completed the self-administered questionnaire; 108 (72%) were females while the rest were males. More than 70% of the study respondents had had more than one surgery, with the majority of the respondents being in the less than 5,000 Riyals income bracket (46%). The average rating of the satisfaction pre-surgery was M (mean) = 8.51, SD (standard deviation) = 1.9 (95% confidence interval (CI) = 8.19 - 8.83), while the average satisfaction rating following surgery was M = 9.05, SD = 1.35 (95% CI = 8.82 - 9.27), both of which were measured on a 10-point scale where high values indicated higher satisfaction. Table 1 gives a summary of the descriptive statistics about the demographic variables of the data.

**Table 1:** Descriptive Statistics SAR: Saudi Arabian Riyal

Variable	Frequency (%)
Age group	
18 - 24	17 (11.3)
25 - 34	21 (14.0)
35 - 44	25 (16.7)
45 - 54	31 (20.7)
55 - 64	29 (19.3)
65 - 74	18 (12.0)
75 or older	9 (6.0)
Gender	
Male	42 (28.0)
Female	108 (72)
First surgery	
No	106 (70.7)
Yes	44 (29.3)
Education level	
Eighth grade or less	48 (32.0)
Some high school	21 (14.0)
High school graduate	30 (20.0)
Two-year college graduate	17 (11.3)
Four-year college graduate	25 (16.7)
Postgraduate	9 (6.0)
Income level	
< 5,000 SAR	69 (46.0)
5,000 - 20,000 SAR	64 (42.7)
> 20,000	17 (11.3)

One hundred and ten of the study respondents (73.3%) said that the specific surgeons who attended to them encouraged them to ask questions. While more than 85% of the respondents said that surgeons or health workers in the department used graphics to explain points, only about 77.5% of these respondents said that the graphics were effective. The proportion of study respondents who said that the surgeon's visit prior to the surgery gave them a calming effect significantly varied across the responses of somewhat, definitely, and no (p < .000). Table 2 presents this information in more detail.

**Table 2:** Pre-surgery Patient/Surgeon Interactions

Variable	Freq. (%)	p-value
Ease of understanding information		.000
Definitely	92 (61.3)	
Somewhat	56 (37.3)	
No	2 (1.3)	
Reason for surgery explained		.000
Adequate	50 (33.3)	
A lot	97 (64.7)	
A little	3 (2.0)	
Reason for no surgery		.000
Adequate	52 (34.7)	
A lot	75 (50.0)	
A little	18 (12.0)	
Not at all	5 (3.3)	
Surgeon spent enough time with the patient		.000
Definitely	97 (64.7)	
Somewhat	51 (34.0)	
No	2 (1.3)	
Patient encouraged to ask questions		.000
Definitely	110 (73.3)	
Somewhat	39 (26.0)	
No	1 (0.7)	
Use of graphics to explain points		.034
Yes	88 (58.7)	
No	62 (41.3)	
Effectiveness of graphics		.000
Definitely	69 (77.5)	
Somewhat	20 (22.5)	
Surgeon visited before surgery		.000
Yes	147 (98.0)	
No	3 (2.0)	
Calming effect of visit		.000
Definitely	97 (64.7)	
Somewhat	43 (28.7)	
No	9 (6)	
Anaesthesiologist administered medicine		.000
Yes	139(92.7)	
Surgeon	4 (2.7)	
Don’t know	7 (4.6)	
Anaesthesiologist encouraged the patient to ask questions		.000
Definitely	79 (52.7)	
Somewhat	54 (36.0)	
No	26 (17.3)	

Most of the respondents (69.3%) said that the surgical team attending to them explained what to expect during recovery, while eight of the respondents (5.3%) disagreed that the surgical team explained to them what to expect after the surgery. The majority of the respondents (76.7%) said that the surgical team gave them definite physical comfort, while the rest (23.3%) said that they got somewhat less physical comfort from the surgical team. All respondents (100%) said that the surgeons contacted them after surgery to check on their recovery. Table 3 gives deeper details of the after-surgery patient experience.

**Table 3:** Post-surgery Health Practitioner/Patient Interaction

Variable	Freq.(%)	p-value
Explanation of expectations during recovery		.000
Definitely	104 (69.3)	
Somewhat	38 (25.3)	
No	8 (5.3)	
Information about warning signs that need medical/surgical attention		.000
Definitely	109 (72.7)	
Somewhat	36 (24.0)	
No	5 (3.3)	
Access to easy to understand instructions		.000
Definitely	108 (72.0)	
Somewhat	36 (24.0)	
No	6 (4.0)	
Physical comfort		.000
Definitely	115 (76.7)	
Somewhat	35 (23.3)	
Contact after surgery with the surgeon		
Yes	150 (100.0)	
Encouraged you to ask questions after surgery		.000
Definitely	112 (74.7)	
Somewhat	31 (20.7)	
No	7 (4.7)	

## Discussion

The main objective of the study was exploring the patient experience before and after surgery for the interactions between the surgical team and the patient's perceived quality of service and care. The results indicated that the average ratings of the pre-surgery experience by the patients were M = 8.51, SD = 1.33, while the average ratings out of a possible 10 after surgery were M = 9.07, SD = 1.33. This is indicative of a positive prospect of the entire surgical process by the patients in terms of quality of care and actual interactions with the surgical team. This finding also resonated with the findings of Al-Mohrej et al., where the general patient-doctor interaction was found to be an important determinant for satisfaction scores before and after surgery [8]. Our survey results point out that the age groups of patients were approximately uniform with no significant difference in the frequencies across the groups (p > 0.05). In comparison, there was a significant difference in the age groups of patients who went for cosmetic surgery in Saudi Arabia where most of the patients were aged between 20 and 40 years (p < 0.05), and most of them reported income brackets more than 5,000 Riyals per month [10-11].

A surgical procedure requires the patient and the surgical team to comprehensively have an exhaustive discussion of the factors of the surgery and life of the patient after the surgical procedure. The high scores reveal how surgical teams ensured that they gave patients easy to understand information, options that did not involve surgical intervention, and so on. Most of the patients were satisfied with the pre-surgical services, with only a minimal number of respondents disagreeing on the quality of patient-doctor interaction. In a study by Jawaid et al., anxiety before elective surgery was reduced by the patient’s perception of understanding what to expect and who to turn to when a matter arises [12]. Surgeon-patient interaction was important in reducing anxiety in the present study, especially when the surgeon visited the patient before surgery. The surgeon’s visit to the patient before surgery, as Kruzik stated, was an important benefit in the form of patient education, especially for elective surgical procedure patients [13].

Our study found that all patients received some form of physical comfort post-surgery at the least. Out of the 150 respondents, 115 said that they received physical comfort, while 35 of the patients said that they somewhat received physical comfort. Additionally, the patients were given information about warning signs that would require medical/surgical attention post-surgery and were given easy to understand instructions (69.3%). This is a reflection of the findings of Meissner et al., which emphasized the importance of contact and constant communication between the surgical team and patients in the management of postoperative surgical procedures [9]. Their findings emphasize a patient-centered focus on the options and methods of management of postoperative care and the eradication of such issues.

This study was a compilation of the responses for patients’ perceptions of the patient-doctor interaction pre- and post-surgery and hence, may not be generalized to an entire population. However, most of the patients were satisfied with the care and human interactions between them and the surgeons and the surgical team members in Saudi Arabia. Only a few cases showed negative responses.

## Conclusions

Most patients value surgeon-patient interaction as it was seen to reduce pre-surgery anxiety and help in giving options and improve the patient’s overall understanding of the surgical procedure. Surgical teams are generally highly rated in terms of overall service pre- and post-surgery.
